# Exercise ventilation and dyspnea in the obese patient with chronic obstructive pulmonary disease: “how much” versus “how well”

**DOI:** 10.1177/14799731211059172

**Published:** 2021-11-26

**Authors:** Jose Alberto Neder

**Affiliations:** Laboratory of Clinical Exercise Physiology and Respiratory Investigation Unit, 4257 Queen’s University & Kingston General Hospital, Kingston, ON, Canada

**Keywords:** Dyspnea, obesity, COPD, lung mechanics, exercise

Dyspnea, usually precipitated or worsened by exertion, is a cardinal symptom of chronic obstructive pulmonary disease (COPD).^1^ Current neurophysiological constructs postulate that there are two main mechanisms by which physical activity may amplify the neural drive to the inspiratory muscles, leading to exertional dyspnea: (a) by increasing the ventilatory demands and (b) by increasing the effort to breathe at a given level of ventilation.^1^ In simple terms, dyspnea depends on “how much” ventilation is required and “how well” (in mechanical terms) such ventilation is achieved, respectively. On a purely descriptive perspective, (a) can be understood as the quantitative domain of exertional dyspnea, whereas (b) reflects its qualitative properties*. A robust body of evidence indicates that the former strongly depends on the extra ventilation required to overcome an enlarged physiological dead space,^2^ while the latter is related to neuromechanical dissociation arising at higher operating lung volumes.^3^ Not surprisingly, therefore, the highest dyspnea scores on exertion are reported by COPD patients depicting (a) poor gas exchange efficiency (i.e., high minute ventilation $\left(\dot{V}E\right)$ -carbon dioxide output $\left(\dot{V}CO_{2}\right)$ relationship) plus (b) critical inspiratory constraints to tidal volume (VT) expansion when they breathe too close to total lung capacity (TLC).^4^

There are several factors, however, that may modulate the severity of exertional dyspnea at a given level of resting functional impairment in patients with COPD.^1^ Amongst them, obesity assumes prominence due to its high prevalence.^5^ Notably, obesity may affect “how much”^6,7^ and “how well”^8^ ventilation is performed on exertion.^9^ For instance, moving a large mass against gravity increases the metabolic cost of work $VCO_{2}$ , leading to higher $\dot{V}E$ , that is, “too much” ventilation.^6^ On the other hand, if functional residual capacity (FRC) is downwardly displaced by the increased weight of the chest wall with little change in TLC, the volume available for VT expansion in the presence of expiratory flow limitation increases, that is, higher inspiratory capacity (IC).^10,11^ Rather paradoxically, therefore, mild-moderate obesity may yield a mechanical advantage to the obese patient with COPD.^12,13^ In fact, this has been previously demonstrated by Ora et al. as the deflating effects of mild-moderate obesity postponed the attainment of critically high inspiratory constraints.^13^ In more severe obesity, however, TLC may decrease, potentially decreasing IC despite a low FRC.^14^ To make things even more complex, fat distribution has a major impact on these inter-relationships since the android/central pattern of obesity has a larger lowering effect on lung volumes (including TLC) compared to the gynecoid/peripheral pattern.^15^ Breathing at excessively low lung volumes, in turn, may predispose to the closure of small airways, leading to ventilation/perfusion mismatch and gas trapping.^16^

In the current issue of *Chronic Respiratory Disease*, Zewari and colleagues^17^ dealt with another feature of the obesitydyspnea conundrum in COPD: the influence of exercise modality. Despite the lack of direct comparisons between walking versus cycling and the dearth of physiological measurements on exertion, the authors conclude that even mild-moderate obesity may have a detrimental effect on exercise tolerance and dyspnea in these patients. Some caution, however, should be exerted to interpret their findings; for instance, only the 6-min walk work (distance walked x weight) was higher in the obese group since there were no between-group differences in the 6-min walking distance. Although statistically significant, 010 Borg dyspnea scores on exercise cessation were only slightly higher in obese subjects (average 4 compared to 3.2). Interestingly, anthropometric markers of abdominal adiposity in the obese group appeared to significantly worsen dyspnea and exercise tolerance, likely due to its greater mechanical consequences.^15^

Why would walking cause greater dyspnea than cycling in the obese patient?^18^ Walking is characteristically associated with greater ventilatory requirements compared to cycling due to (a) higher $\left(\dot{V}CO_{2}\right)$ on weight-bearing compared to weight-supported exercise,^6^ (b) additional ventilatory stimuli secondary to upper limbs’ movement,^9^ and, in some patients, (c) greater exercise-induced hypoxemia resulting from lower mixed venous O_2_ pressures and a delayed onset of hyperventilation to compensate for metabolic acidosis.^19^ The abdominal expiratory muscles are involved in postural actions during walking; being less available to decrease FRC and increase IC compared to cycling.^8^ If the patients anchor their accessory inspiratory muscles by firmly holding the handlebars, larger IC can be reached on cycling than walking.^20^ Zewari and colleagues^17^ argue that the negative consequences of mild-moderate obesity on dyspnea during walking have more than eclipsed its putative salutary effects previously described by Ora et al. in response to cycling.^3^ As mentioned, however, these assertions were based on indirect comparisons between different populations. Dynamic hyperinflation was only inferred by changes in IC induced by short (20 s) increases in resting breathing frequency at rest, that is, it was not measured during exercise. Moreover, other mechanisms of exercise intolerance which are more prevalent in the obese, such as severe deconditioning and cardiovascular co-morbidities, may have varied substantially in each individual study.

There remains, therefore, several unanswered questions on this topic. For instance, it is conceivable that the negative consequences of increased ventilatory demands associated with walking are particularly pronounced in patients with higher “wasted” ventilation in the physiological dead space.^2^ Is there an equilibrium point where the putative beneficial effects of mild-moderate obesity on lung mechanics compensate for the negative consequences of increased ventilatory demands? If so, this is likely to vary with exercise intensity and whether there are other sources of ventilation stimuli, such as a low PaO_2_ or early metabolic acidosis, or not. Are the negative effects of android/central obesity on exertional dyspnea greater on walking than cycling? Are they even greater in subjects with small trunks, that is, shorter patients? Importantly, most studies looked at patients with mild-moderate obesity with only a few patients in the morbid obesity range.

To answer these and other questions,^21^ future studies should consider not only the severity of obesity as estimated by body mass index (BMI) but, crucially, body fat distribution in a sizable number of men and women showing a large range of resting functional abnormalities. The same subjects should undergo walking and cycling exercise: their sensory and physiological responses (including operating lung volumes) compared at iso-work rate and iso-ventilation. Ideally, measurements should be repeated after weight loss to determine the directional changes on the quantitative and qualitative domains of exertional dyspnea.^22^ Advancing the knowledge on the seeds and consequences of activity-related dyspnea in the obese patient with COPD are likely to directly impact the care of this ever-growing patient subpopulation.^5^
